# A 1 V 92 dB *SNDR* 10 kHz Bandwidth Second-Order Asynchronous Delta-Sigma Modulator for Biomedical Signal Processing

**DOI:** 10.3390/s20154137

**Published:** 2020-07-25

**Authors:** Vilém Kledrowetz, Lukáš Fujcik, Roman Prokop, Jiří Háze

**Affiliations:** Department of Microelectronics, Brno University of Technology (BUT), Technická 3058/10, 61600 Brno, Czech Republic; fujcik@vutbr.cz (L.F.); prokopr@vutbr.cz (R.P.); haze@vutbr.cz (J.H.)

**Keywords:** asynchronous delta-sigma modulator (ADSM), center frequency, operational amplifier, biomedical signals, biosensors

## Abstract

In this paper, a second-order asynchronous delta-sigma modulator (ADSM) is proposed based on the active-RCintegrators. The ADSM is implemented in the 0.18 μm CMOS Logic or Mixed-Signal/RF, General Purpose process from the Taiwan Semiconductor Manufacturing Company with a center frequency of 848 kHz at a supply voltage of 1 V with a 92 dB peak signal-to-noise and distortion ratio (SNDR), which corresponds to 15 bit resolution. These parameters were achieved in all the endogenous bioelectric signals bandwidth of 10 kHz. The ADSM dissipated 295 μW and had an area of 0.54 mm${}^{2}$. The proposed ADSM with a high resolution, wide bandwidth, and rail-to-rail input voltage range provides the universal solution for endogenous bioelectric signal processing.

## 1. Introduction

**Figure 1:**
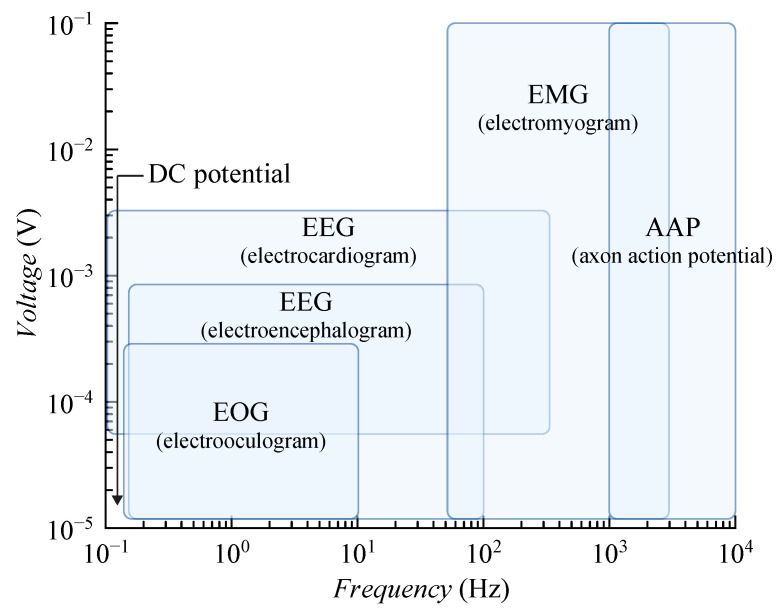
Voltage and frequency ranges of some common biopotential signals.

Biomedical electronics have acquired significant attention in healthcare, with a focus on the development of biosensors that enable online monitoring, detection, prevention, and personalized medicine for a variety of chronic and acute diseases. Especially in the last few years, there has been growing interest in the design of biomedical wireless sensors [1,2,3]. Biomedical signals can be subdivided into two major classes: (1) endogenous signals that arise from natural physiological processes and are measured within or on living creatures (e.g., (EOG), electroencephalogram (EEG), electrocardiogram (ECG or EKG), electromyogram (EMG), temperature, blood glucose, etc.) and (2) exogenous signals applied from the outside (generally noninvasively) to measure internal structures and parameters. Endogenous bioelectric signals are invariably small, ranging from single microvolts to over 100 mV. Their bandwidths range from DC to perhaps 10 kHz at most [4,5,6,7]. The voltage and frequency ranges of some common biopotential signals are shown in Figure 1.

A general biomedical system consists of an energy source, a differential amplifier, analog-to-digital conversion (ADC), digital signal preprocessing, and a communication subsystem. The ADC is one of the key building blocks, which enables converting analog signals from biomedical sensors to a digital format that can be easily processed and analyzed. For the design of the ADC, many authors choose the SARarchitecture due to its suitability for low-power and low-voltage requirements [8,9,10,11]. Recently, delta-sigma (ΔΣ) ADC has been gaining more and more popularity. Compared to other conversion techniques, ΔΣ ADCs cover the widest conversion region of the resolution-versus-bandwidth plane, providing the most efficient solution to digitize diverse types of signals in many different applications such as biomedical ones [12]. There exist two basic types of ΔΣ modulators: discrete-time (DTDSM) and continuous-time (CTDSM). The DTDSM is more attractive for high-resolution applications due to its higher linearity and accuracy. On the other hand, less stringent amplifier speed specifications are required in CTDSM due to the absence of switches in the active-RC integrator, allowing achieving a higher speed of operation and lower power consumption. The asynchronous ΔΣ modulator (ADSM) can be considered as a special type of CTDSM. ADSM is simple, does not require any clocking, matches well with mainstream CMOS technology, and can operate at low current and supply voltages [13,14,15]. A comparison between DTDSM, CTDSM, and ADSM is shown in Table 1.

In several publications, DTDSMs are used for biomedical signal processing [1,16,17]. There also exists solutions utilizing ADSM [18,19,20]. These ADSM are distinguished by very low power consumption in the order of tens of nanowatts. However, their bandwidth is very low in the order of tens of Hertz. The proposed ADSM covers the full bandwidth of endogenous bioelectric signals up to 10 kHz. The differential input range equals $V_{DDA}$ with a 0.5 V reference level ($V_{CM}$). The proposed ADSM with high resolution, wide bandwidth, and rail-to-rail input voltage range provides the universal solution for endogenous bioelectric signal processing. The circuit not only offers an alternative to the developed CTDSMs and DTDSMs, but it also fills the gap between published ADSMs, which do not allow processing the full spectrum of biomedical signals according to Figure 1 except for those with a very high bandwidth in the order of MHz. An important parameter of ADSM is the center frequency, the calculation of which is part of this work. The following sections provide the details of our approach.

## 2. Asynchronous Delta-Sigma Modulator

There are two major types of architecture for ΔΣ modulators. The first one is the single-loop and the second the multi-loop architecture. Multi-loop architectures are commonly denoted as cascade or MASH (multi-stage noise shaping). A major drawback of MASH modulators is that precise matching of the analog and digital signal processing paths is required to avoid large errors (quantization noise leakage) caused by integrator gain coefficient variations. Because RC integrators are used in this design, where variations of about 20% in the RC time constant can be expected, the single-loop architecture was chosen in this work. Its stronger ability to achieve high *SNDR* since it does not suffer from matching errors, which severely affect MASH modulators, is the major advantage in the design.

**Figure 2:**
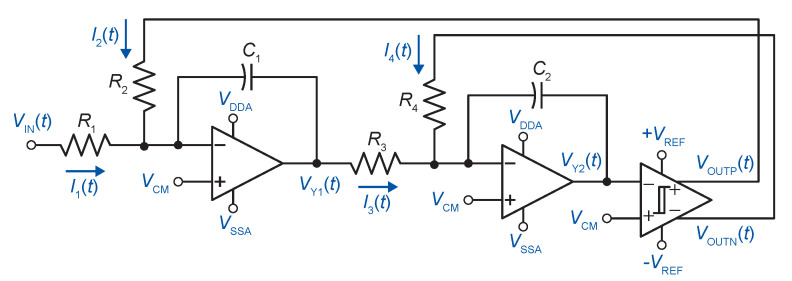
Simplified schematic of the second-order ADSM with the CIFB topology.

The block diagram of the second-order ADSM based on the cascade of integrators with distributed feedback (CIFB) topology is shown in Figure 2.

The circuit consists of two integrators and a binary quantizer with hysteresis. The output $V_{OUTP}$ (or $V_{OUTN}$) is a pulse width modulated square wave of period $T_{PER}$ with a pulse width $T_{PW}$. The duty cycle *d* is proportional to the amplitude of the input signal (Equation (1)). Moreover, the period $T_{PER}$ of the asynchronous modulator output signal is modulated by the normalized input voltage $V_{IN}$ (Equation (2)) [21].

(1) $$d=\frac{V_{IN}+1}{2}=\frac{T_{PW}}{T_{PER}}$$

and:

(2) $$\frac{f_{0}}{f_{c}}=1-V_{IN}^{2}\;and\;\left|v\right|<1$$

where $f_{0}$ is the output carrier frequency, $f_{c}$ is the maximum value of $f_{0}$, namely the center frequency, and $\left|V_{IN}\right|<1$ is the normalized input amplitude.

The center frequency of ADSMs determines the carrier-to-bandwidth ratio ($CBR=f_{c}/\left(2B\right)$, where *B* is the input signal bandwidth), which is the ratio between the center frequency and the signal bandwidth. This ratio is equal to the oversampling ratio (OSR) in synchronous delta-sigma modulators. It determines the minimal center frequency required for a certain conversion accuracy. The critical condition can occur when $V_{IN}$ is close to the full scale. The output frequency will decrease, and the high-frequency distortions around the center frequency shift to the low-frequency region. Consequently, distortions can leak into the baseband and adversely affect the modulator linearity for large input amplitudes. Therefore, the center frequency should be set far away from the baseband to avoid these components shifting into the signal baseband, and a high order filter is required to attenuate these out-band components. In order to achieve a high center frequency without degeneration of the linearity, the second-order topology was chosen. To calculate the center frequency of the proposed ADSM, the integrators’ output voltages are expressed as:

(3) $$V_{Y1}\left(t\right)=-\left(\frac{I_{1}\left(t\right)}{C_{1}}+\frac{I_{2}\left(t\right)}{C_{1}}\right)t+V_{CM}$$

(4) $$V_{Y2}\left(t\right)=-\left(\frac{I_{3}\left(t\right)}{C_{2}}+\frac{I_{4}\left(t\right)}{C_{2}}\right)t+V_{CM}$$

where $I_{1}\left(t\right)$, $I_{2}\left(t\right)$, $I_{3}\left(t\right)$, and $I_{4}\left(t\right)$ can be expressed as:

(5) $$I_{1}\left(t\right)=\frac{V_{IN}\left(t\right)-V_{CM}}{R_{1}}$$

(6) $$I_{2}\left(t\right)=\frac{V_{REF}\left(t\right)-V_{CM}}{R_{2}}$$

(7) $$I_{3}\left(t\right)=\frac{V_{Y1}\left(t\right)-V_{CM}}{R_{3}}$$

(8) $$I_{4}\left(t\right)=\frac{V_{REF}\left(t\right)-V_{CM}}{R_{4}}$$

**Figure 3:**
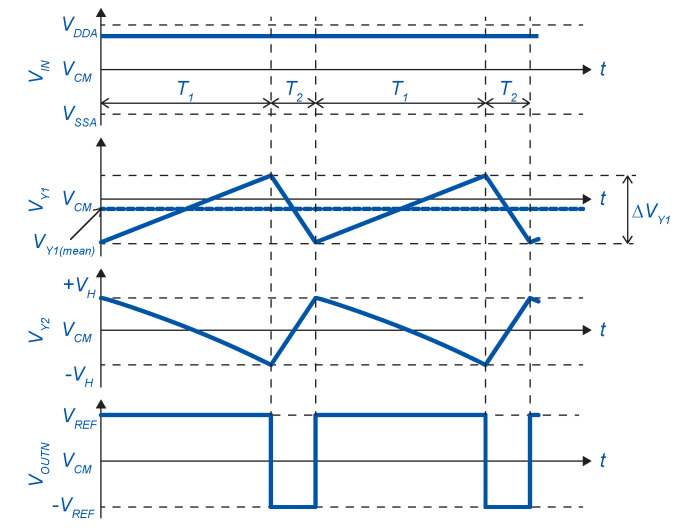
Timing diagram of the asynchronous sigma delta modulator with a constant input.

In order to find the center frequency, the timing diagram in Figure 3 is considered, which corresponds to the schematic in Figure 2.

The duty cycle of the ADSM is given by the ratio of rising ($S_{RE}$)-to-falling edge ($S_{FE}$) speed. To facilitate the equations, a symmetrical power supply is considered ($V_{DDAs}=V_{DDA}-V_{CM}$, $V_{CM}$ = 0 V, $V_{SSAs}=V_{SSA}-V_{CM},V_{REF}=V_{DDAs}=\left|V_{SSAs}\right|;\left|V_{HL}-V_{CM}\right|=V_{HH}-V_{CM}=V_{H}$). During the $T_{1}$ period, the output voltage of the first integrator $V_{Y1}$ rises with speed, given by:

(9) $$S_{RE1}=\frac{dV_{Y1}}{dt}=\frac{I_{1}+I_{2}}{C_{1}}=\frac{V_{IN}R_{2}+V_{REF}R_{1}}{R_{1}R_{2}C_{1}}$$

and the falling edge during $T_{2}$:

(10) $$S_{FE1}=\frac{dV_{Y1}}{dt}=\frac{I_{1}+I_{2}}{C_{1}}=\frac{V_{IN}R_{2}-V_{REF}R_{1}}{R_{1}R_{2}C_{1}}$$

The first integrator output voltage swing is in the range of:

(11) $$\Delta V_{Y1}=V_{Y1(mean)}\pm \left|V_{H}\right|\frac{I_{2}}{I_{4}}$$

where $V_{H}$ is the comparator threshold voltage.

In order to find $V_{Y1(mean)}$, we calculate $I_{3(mean)}$. The duty cycle of the second integrator output $V_{Y2}$ is the same as the first one. From Equations (9) and (10), the value of $I_{3(mean)}$ is calculated to meet the duty cycle requirements.

(12) $$I_{3(mean)}=\frac{V_{Y1(mean)}}{R_{3}}=-\frac{V_{IN}R_{2}}{R_{1}R_{4}}$$

For period $T_{1}$, we can write:

(13) $$T_{1}=\frac{2V_{H}R_{1}R_{4}C_{2}}{R_{1}V_{REF}-V_{IN}R_{2}}$$

(14) $$T_{2}=\frac{2V_{H}R_{1}R_{4}C_{2}}{R_{1}V_{REF}+V_{IN}R_{2}}$$

The entire period can be expressed as:

(15) $$T_{PER}=T_{1}+T_{2}$$

When a zero input is applied $V_{IN}=0$, the output of the ADSM is a square wave with a duty cycle of 50%. By defining $T_{C}$ as the period of the output signal, it can be calculated as:

(16) $$T_{C}=T_{PER}=2T_{1}=2T_{2}=\frac{4V_{H}R_{4}C_{2}}{V_{REF}}$$

Similar to the conventional synchronous CTDSMs, propagation delay is also an issue in ADSMs. The delay of the comparator increases the effective value of hysteresis and negligibly affects the center frequency of the ADSM. Therefore, the impact of the comparator delay, τ, on the center frequency of the proposed ADSM can be given by:

(17) $$T_{C}=T_{PER}=2T_{1}=2T_{2}=\frac{1}{f_{C}}=\frac{4V_{H}R_{4}C_{2}}{V_{REF}}+\tau$$

Equation (17) shows that the center frequency $f_{C}$ of the modulator will decrease for a higher delay of the comparator, which degenerates the input bandwidth and linearity of the modulator [20].

## 3. Transistor Level Realization

**Figure 4:**
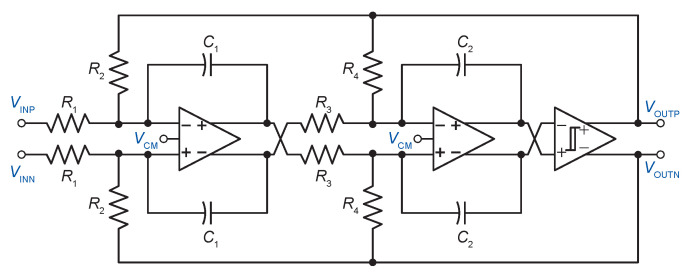
Proposed second-order asynchronous delta–sigma modulator.

In this section, the transistor level implementation of the ADSM will be described. Figure 4 illustrates the circuit diagram of the proposed ADSM. The implemented architecture is fully differential to minimize even-order harmonics, as well as common-mode noise.

### 3.1. Active-RC Integrator

In the proposed design, the active-RC integrators were used due to simplicity, high linearity, parasitic insensitivity, as well as the overall power consumption. The ideal transfer function of the active-RC integrator is given by:

(18) $$Int\left(s\right)=\frac{1}{sRC}=k_{i}\frac{f_{s}}{s}$$

where $f_{s}$ is the sampling frequency and $k_{i}$ is the scaling coefficient.

**Figure 5:**
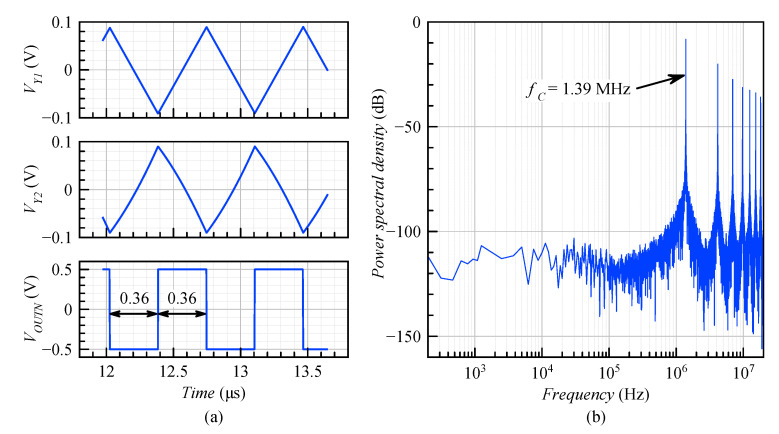
MATLAB model simulation results: (**a**) timing diagram and (**b**) frequency spectrum of the ADSM for $V_{IN}$ = 0 V.

The parameters of the resistors and capacitors were designed to achieve a high center frequency according to Equation (16). The lower limit for *R* and *C* is defined by matching consideration and maximum charging current in the case of *R*. The upper limit for *R* is set by the allowed thermal noise level, which itself is fixed by the overall dynamic range requirements. Finding optimal *R* and *C* was also confirmed by behavioral simulations in MATLAB/Simulink, as well as variations of about 20% in the RC time constant. The *R* and *C* values were $R_{1}$ = 650 kΩ, $R_{2}$ = $R_{4}$ = 500 kΩ, $R_{3}$ = 357 kΩ, $V_{H}$ = 90 mV, $C_{1}$ = $C_{2}$ = 2 pF, and $I_{R}$ = 1 μA. It can be calculated from Equation (16) that $f_{C}$ = 1.39 MHz, and from Equation (11), $V_{Y1(mean)}$ = (0 ± 90) mV. The validity of these results was verified in MATLAB/Simulink and is shown in Figure 5.

As will be seen later, nonidealities such as input parasitic capacitances of the operational amplifier and the delay of the comparator negligibly affect the center frequency of the modulator.

### 3.2. Class AB Fully Differential Operational Amplifier

**Figure 6:**
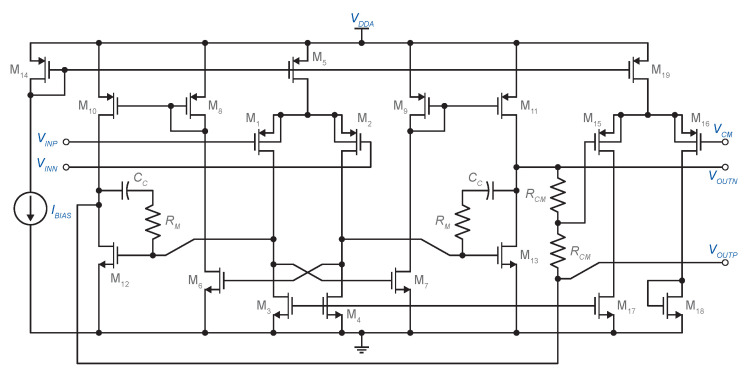
Circuit schematic of the two-stage Class A/AB operational amplifier.

The integrators in the ADSM were each implemented using the two-stage, Class A/AB operational amplifier topology shown in Figure 6. This topology combines a simple differential pair as the first stage with a Class A/AB second stage, wherein push-pull operation is implemented using current mirrors [22]. The slew-rate is limited only by the first stage.

(19) $$SR=\frac{I_{5}}{C_{C1}+C_{G6}+C_{G13}}$$

The minimum value of the operational amplifier slew-rate can be determined from the falling edge speed of $V_{Y2}$ ($S_{FE2}$) according to Figure 3. The input parasitic capacitance of the comparator ($C_{comp}$) should be included in the calculations. Thus,

(20) $$\left|S_{FE2}\right|=\frac{dV_{Y2}}{dt}=\frac{I_{3}+I_{4}}{C_{2}+C_{comp}}=\frac{V_{Y1}R_{4}+V_{REF}R_{3}}{R_{3}R_{4}\left(C_{2}+C_{comp}\right)}$$

The use of PMOS input transistors makes it possible to avoid the body effect. The compensation network is comprised of capacitor $C_{C}$ and resistor $R_{M}$, which cancels the right half plane zero.

For the detection of the common-mode output voltage, two equal resistors were used ($R_{CM}=500$ kΩ). The voltage between the two resistors is subtracted from the desired common-mode output voltage, $V_{CM}$, and scaled by the one-stage differential amplifier that consists of source-coupled pair M${}_{15}$–M${}_{16}$, diode-connected loads M${}_{17}$ and M${}_{18}$, and tail current source M${}_{19}$. The main reason for using this solution is that the input to the common-mode sense amplifier (gate of M${}_{15}$) is almost constant. Therefore, this CMFB solution does not limit the operational amplifier output voltage swing. Table 2 sums up the simulated parameters of the operational amplifier used in the integrators.

### 3.3. Comparator with Hysteresis

**Figure 7:**
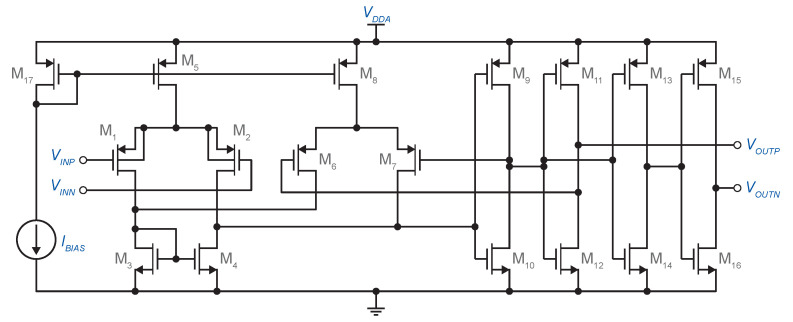
Comparator with hysteresis using an unbalanced differential pair.

The schematic of a comparator using the internal positive feedback circuit is given in Figure 7. The comparator consists of a differential pair (M${}_{1}$–M${}_{2}$) with output inverters in order to to achieve reasonable voltage swings, output resistance, and differential output. A second, smaller differential pair, M${}_{6}$–M${}_{7}$, unbalances the input differential pair. The inputs of the second differential pair are tied to the output signals in such a way as to introduce positive feedback and, hence, hysteresis.

If M${}_{1}$ and M${}_{2}$ are operating in strong inversion, the amount of hysteresis $V_{TH}-V_{TL}$ can be calculated using [23]:

(21) $$V_{TH}-V_{TL}=\frac{2(\sqrt{I_{D3}+I_{D11}}-\sqrt{I_{D3}-I_{D11}})}{\sqrt{\mu C_{OX}{\left(W/L\right)}_{1}}}$$

If M${}_{1}$ and M${}_{2}$ are operating in weak inversion,

(22) $$V_{TH}-V_{TL}=4nU_{T}\tanh^{-1}\left(I_{D11}/I_{D3}\right)$$

Assume that the gate of M${}_{1}$ ($V_{INP}$) is tied to $V_{DDA}$. With the input of M${}_{2}$ ($V_{INN}$) much less than $V_{DDA}$, M${}_{1}$ is off and M${}_{2}$ on, and $V_{OUTP}$ is at $V_{DDA}$ and $V_{OUTN}$ at $V_{SSA}$, thus turning on M${}_{7}$ and turning off M${}_{4}$ and M${}_{6}$. In this state, no current flows through the differential pairs. As the voltage at the $V_{INP}$ input decreases toward the threshold point (Equations (21) or (22)), some of $I_{D5}$ begins to flow through M${}_{1}$ and M${}_{3}$, and simultaneously, some of the hysteresis bias current $I_{D8}$ begins to flow through M${}_{4}$. This continues until the point where the current through M${}_{1}$ equals the current $I_{D8}$. Just beyond this point, the comparator switches its state.

**Figure 8:**
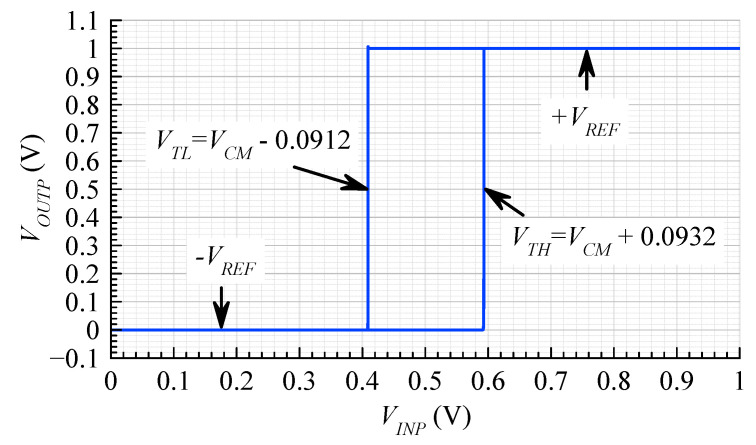
Simulated DC transfer characteristic of the comparator.

Figure 8 shows the voltage transfer characteristic of the comparator. The hysteresis bias current $I_{D8}$ was 6.8 μA, and the input bias current $I_{D5}$ was 10 μA. The output high-to-low threshold, $V_{TL}$, was simulated as −90 mV. The output low-to-high threshold, $V_{TH}$, was simulated as +90 mV. The amount of hysteresis was 180 mV. Simulated parameters of the comparator circuit are given in Table 3.

According to parameters mentioned in Table 2 and Table 3, the center frequency was recalculated to $f_{C}=857$ kHz.

## 4. Simulation Results

**Figure 9:**
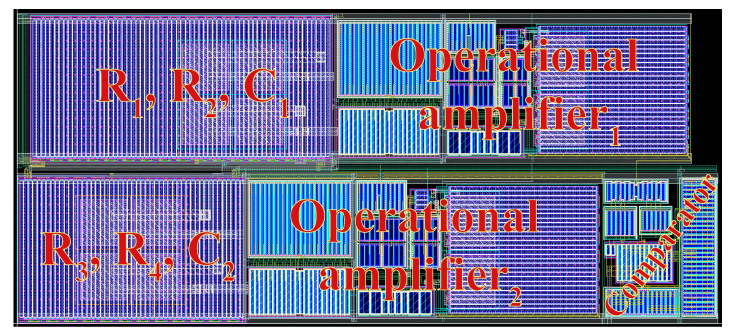
Layout of the proposed ADSM.

The ADSM was designed utilizing the 0.18 μm CMOS Logic or Mixed-Signal/RF, General Purpose process from the Taiwan Semiconductor Manufacturing Company. The circuit was designed for $V_{DD}$ = 1 V and $I_{BIAS}$ = 2.5 μA. After completion of the layout design, its parasitic extraction was performed to find the parasitic resistances and capacitances corresponding to the designed devices and interconnects. After parasitic extraction, all simulations were performed using the Spectre simulator on the Cadence platform. The layout of the ADSM is shown in Figure 9. The layout size is 350 × 155 μm.

**Figure 10:**
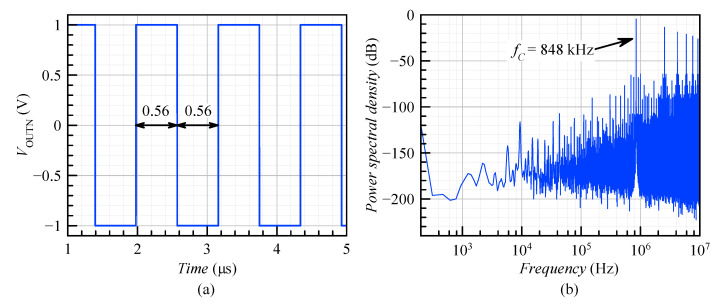
Post-layout simulation results: (**a**) timing diagram and (**b**) frequency spectrum of the ADSM for $V_{IN}$ = 0 V.

The ADSM output bitstream can be recovered by applying an ideal low pass filter with a cut-off frequency at the signal bandwidth. When ADSMs are used in A/D data conversion, a decoding circuit is required. The simplest one is the sample and hold circuit with a high sampling frequency. The time domain waveforms of the output signal $V_{OUTN}$ for $V_{IN}$ = 0 V and the corresponding frequency spectrum are shown in Figure 10. The limit cycle frequency of the post-layout model of the ADSM was equal to 848 kHz and was very close to the calculated value in Section 3.3 ($f_{C}=857$ kHz). The small difference was caused by the parasitic capacitances and resistances extracted from the layout.

**Figure 11:**
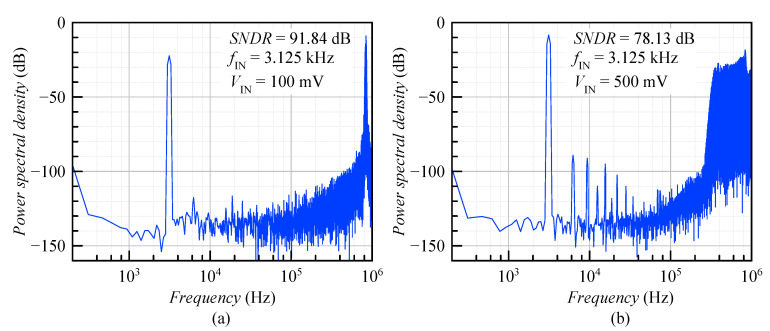
PSDof the ADSM for the sinusoidal input signal with an amplitude of (**a**) $V_{IN}$ = 100 mV and (**b**) $V_{IN}$ = 500 mV.

Figure 11 shows the simulated spectrum of the ADSM for a sinusoidal input signal with an amplitude of (a) 100 mV (20% modulation depth) and (b) 500 mV (100% modulation depth). The corresponding spectra were obtained by applying a signal at $f_{IN}\leq f_{Bandwidth}/3$ to include at least the second and third harmonic inside the band of interest. Due to this reason, the input frequency was set to 3.125 kHz, and then the third harmonic component was located in the 10 kHz bandwidth. The achieved SNDR was (a) 91.84 dB and (b) 78.13 dB. In the second case, the significant SNDR reduction was caused by higher harmonic tones.

**Figure 12:**
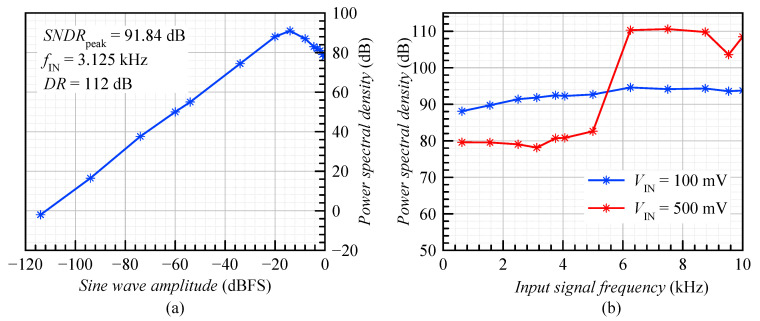
Plot of the SNDR vs. the (**a**) input sine wave amplitude and (**b**) input signal frequency.

Figure 12a shows the simulated dynamic range (DR) with respect to the amplitude of the input signal with a frequency of both 3.125 kHz and 6.25 kHz. As the sine wave amplitude increased, the SNDR increased to reach the peak of 91.84 dB at −14 dBFS, and then dropped to 78.13 dB at 0 dBFS.

Figure 12b shows the SNDR vs. input signal frequency with an amplitude of both 100 mV and 500 mV. In the case of the input sine wave amplitude of 100 mV, the SNDR was above 88 dB for all frequencies up to the 10 kHz bandwidth. In the second case, the SNDR dropped from 110 dB to below 80 dB at frequencies smaller than 5 kHz ($f_{Bandwidth}/2$). This was because the second harmonic penetrated into the baseband and significantly reduced the SNDR. The dynamic range was equal to 112 dB.

Table 4 presents a comparison of the proposed ADSM with other DSMs, which are capable of processing endogenous bioelectric signals in the full frequency range. Two figures of merit ($FOM_{1}$, $FOM_{2}$) are defined in Equations (23) and (24) for a better comparison. The first one emphasizes power consumption, whereas the second one emphasizes resolution. A better performance of DSMs is indicated by smaller $FOM_{1}$ and larger $FOM_{2}$ values.

(23) $$FOM_{1}=\frac{P_{cons}}{2^{ENOB}2BW}$$

(24) $$FOM_{2}=DR+10\log\frac{BW}{P_{cons}}$$

As can be concluded from Table 4, the proposed modulator offers a high SNDR and the best values of $FOM_{2}$. Higher power consumption could be further improved utilizing a one-stage operational amplifier with much lower power consumption. The most attractive feature of ADSMs is the simple circuit architecture and clock-less operation. This feature can be very useful in applications in wireless sensors, where a decoding circuit (e.g., time-to-digital converter) is realized outside the integrated circuit.

## 5. Conclusions

This paper presents a second-order ADSM utilizing active-RC integrators. The circuit was designed in the 0.18 μm CMOS Logic or Mixed-Signal/RF, General Purpose process from the Taiwan Semiconductor Manufacturing Company. Post-layout simulation was performed using the Spectre simulator on the Cadence platform. The proposed ADSM with a center frequency of 828 kHz achieves a 92 dB peak SNDR, while having a Walden FOM of 0.45 pJ/step and a Shreirer FOM of 187 dB. These parameters together with a bandwidth of 10 kHz provide the universal solution for endogenous bioelectric signal processing. The overall power consumption is 295 μW, while the chip area corresponds only to 0.54 mm${}^{2}$.

## Figures and Tables

**Table 1:** Comparison between DTDSM, CTDSM, and ADSM.

DTDSM	CTDSM	ADSM
+ Synchronous system	+ Synchronous system	+ Immunity to clock jitter
+ High resolution	+ Implicit antialiasing filter	+ Implicit antialiasing filter
+ Highly linear SC integrator	+ Higher sampling frequency	+ Simple circuit
+ Accurately defined integrator	+ Relaxed operational amplifier	+ Relaxed OpAmp
gains and transfer function	speed requirements	speed requirements
+ Low sensitivity to clock jitter	+ Higher conversion speed	+ Higher conversion speed
and excess loop delay	+ Low power	+ Low power
+ Low sensitivity to DAC settling time		+ Do not require a clock
- Low conversion speed	- Sensitivity to clock jitter	- Complex decoding scheme
- Required pre-antialiasing filter	- Excess loop delay	- Lack of noise shaping
- Required non-overlapping	- Lower resolution	- Lower resolution
clock generator		

**Table 2:** Simulated parameters for the operational amplifier ($C_{L}$ = 3 pF).

Parameter	Condition	Value
Hysteresis	$\left|V_{TH}\right|=\left|V_{TL}\right|$	90 mV
Time delay	$C_{L}=3\mathrm{pF}$	50 ns
Slew-rate	$C_{L}=3\mathrm{pF}$	42 V/μs
Power consumption	duty cycle = 50%	5 μW
Input capacitance		0.4 pF
$I_{BIAS}$		2.5 μA

**Table 3:** Simulated parameters for the comparator.

Parameter	Condition	Value
Hysteresis	$\left|V_{TH}\right|=\left|V_{TL}\right|$	90 mV
Time delay	$C_{L}=3\;\mathrm{pF}$	50 ns
Slew-rate	$C_{L}=3\;\mathrm{pF}$	42 V/μs
Power consumption	duty cycle = 50%	5 μW
Input capacitance		0.4 pF
$I_{BIAS}$		2.5 μA

**Table 4:** Specifications of the proposed ADSM in comparison with other DSMs.

Parameter	This Work	[24] 2015	[25] 2016	[26] 2018	[27] 2012
Technology	180 nm	130 nm	65 nm	65 nm	130 nm
Topology	ADSM	DTDSM	CTDSM	DTDSM	DTDSM
Order	2	3	3	3 (2-1 MASH)	3
Quantizer	1 bit	1 bit	5 bit	1 bit	1 bit
Supply voltage	1 V	0.4 V	1 V	1 V	0.25 V
Modulator frequency	848 kHz	3.2 MHz	6.4 MHz	5 MHz	1.4 MHz
Bandwidth	10 kHz	20 kHz	25 kHz	25 kHz	10 kHz
Peak SNDR	92 dB	76.1 dB	95.2 dB	94.6 dB	61 dB
Dynamic range	112 dB	82 dB	103 dB	98.5 dB	≅ 58 dB
Power consumption	290 μW	63 μW	800 μW	175 μW	7.5 μW
Area	0.54 mm${}^{2}$	0.33 mm${}^{2}$	0.256 mm${}^{2}$	0.384 mm${}^{2}$	0.3375 mm${}^{2}$
$FOM_{1}$	0.45 pJ/step	0.31 pJ/step	0.34 pJ/step	0.079 pJ/step	0.41 pJ/step
$FOM_{2}$	187 dB	167 dB	177.9 dB	176.2 dB	-

## Abbreviations

The following abbreviations are used in this manuscript:

ADC	Analog-to-digital converter
ADSM	Asynchronous delta-sigma modulator
CBR	Carrier-to-bandwidth ratio
CIFB	Cascade of integrators with distributed feedback
CTDSM	Continuous-time delta-sigma modulator
DR	Dynamic Range
DTDSM	Discrete-time delta-sigma modulator
FOM	Figure-of-merit
MASH	Multi-stage noise shaping
SNDR	Signal-to-noise and distortion ratio

## References

[1] Kledrowetz V., Prokop R., Fujcik L., Pavlik M., Háze J. (2019). Low-power ASIC suitable for miniaturized wireless EMG systems. J. Electr. Eng..

[2] Magno M., Benini L., Spagnol C., Popovici E. Wearable low power dry surface wireless sensor node for healthcare monitoring application. Proceedings of the 2013 IEEE 9th International Conference on Wireless and Mobile Computing, Networking and Communications (WiMob).

[3] Vinod A.P., Da C.Y. An integrated surface EMG data acquisition system for sports medicine applications. Proceedings of the 2013 7th International Symposium on Medical Information and Communication Technology (ISMICT).

[4] Northrop R.B. (2004). Analysis and Application of Analog Electronic Circuits to Biomedical Instrumentation (Biomedical Engineering).

[5] Webster J. (2010). Medical Instrumentation: Application and Design.

[6] Prutchi D., Norris M. (2005). Design and Development of Medical Electronic Instrumentation.

[7] Delgado J.M. (1964). Electrodes for extracellular recording and stimulation. Electrophysiological Methods.

[8] Rodríguez-Pérez A., Delgado-Restituto M., Medeiro F. Power Efficient ADCs for Biomedical Signal Acquisition. http://citeseerx.ist.psu.edu/viewdoc/download?doi=10.1.1.936.432&rep=rep1&type=pdf.

[9] Zou X., Xu X., Yao L., Lian Y. (2009). A 1-V 450-nW Fully Integrated Programmable Biomedical Sensor Interface Chip. IEEE J. Solid-State Circuits.

[10] Sundarasaradula Y., Constandinou T.G., Thanachayanont A. A 6-bit, two-step, successive approximation logarithmic ADC for biomedical applications. Proceedings of the 2016 IEEE International Conference on Electronics, Circuits and Systems (ICECS).

[11] Mesgarani A., Ay S.U. A low voltage, energy efficient supply boosted SAR ADC for biomedical applications. Proceedings of the 2011 IEEE Biomedical Circuits and Systems Conference (BioCAS).

[12] De la Rosa J.M., Schreier R., Pun K., Pavan S. (2015). Next-Generation Delta-Sigma Converters: Trends and Perspectives. IEEE J. Emerg. Sel. Top. Circuits Syst..

[13] Ouzounov S., Engel R., Hegt J.A., van der Weide G., van Roermund A.H.M. (2006). Analysis and design of high-performance asynchronous sigma-delta Modulators with a binary quantizer. IEEE J. Solid-State Circuits.

[14] Dazhi W., Vaibhav G., Harris J.G. An asynchronous delta-sigma converter implementation. Proceedings of the 2006 IEEE International Symposium on Circuits and Systems.

[15] Daniels J., Dehaene W., Steyaert M.S., Wiesbauer A. (2010). A/D conversion using asynchronous delta-sigma modulation and time-to-digital conversion. IEEE Trans. Circuits Syst. I Regul. Pap..

[16] Lee J., Song S., Roh J. (2019). A 103 dB DR Fourth-Order Delta-Sigma Modulator for Sensor Applications. Electronics.

[17] Sohel A., al Khadir A., Naaz M., Najeeb A. A 1.8V 204.8-μW 12-Bit Fourth Order Active Passive ΣΔ Modulator for Biomedical Applications. Proceedings of the 2019 Devices for Integrated Circuit (DevIC).

[18] Ferreira L.H.C., Sonkusale S.R. (2015). A 0.25-V 28-nW 58-dB Dynamic Range Asynchronous Delta Sigma Modulator in 130-nm Digital CMOS Process. IEEE Trans. Very Large Scale Integr. (VLSI) Syst..

[19] Kulej T., Khateb F., Ferreira L.H.C. (2019). A 0.3-V 37-nW 53-dB SNDR Asynchronous Delta–Sigma Modulator in 0.18-μm CMOS. IEEE Trans. Large Scale Integr. (VLSI) Syst..

[20] Akbari M., Hashemipour O., Moradi F. (2017). Design and analysis of an ultra-low-power second-order asynchronous delta–sigma modulator. Circuits Syst. Signal Process..

[21] Roza E. (1997). Analog-to-digital conversion via duty-cycle modulation. IEEE Trans. Circuits Syst. II Analog Digit. Signal Process..

[22] Rabii S., Wooley B.A. (1997). A 1.8-V digital-audio sigma-delta modulator in 0.8-/spl mu/m CMOS. IEEE J. Solid-State Circuits.

[23] Furth P.M., Tsen Y.C., Kulkarni V.B., Raju T.K.P.H. (2010). On the design of low-power CMOS comparators with programmable hysteresis. Proceedings of the 2010 53rd IEEE International Midwest Symposium on Circuits and Systems.

[24] Yoon Y., Choi D., Roh J. (2015). A 0.4 V 63 μW 76.1 dB SNDR 20 kHz Bandwidth Delta-Sigma Modulator Using a Hybrid Switching Integrator. IEEE J. Solid-State Circuits.

[25] Leow Y.H., Tang H., Sun Z.C., Siek L. (2016). A 1 V 103 dB 3rd-Order Audio Continuous-Time ΔΣ ADC with Enhanced Noise Shaping in 65 nm CMOS. IEEE J. Solid-State Circuits.

[26] Liao S., Wu J. A 1 V 175 μW 94.6 dB SNDR 25 kHz bandwidth delta-sigma modulator using segmented integration techniques. Proceedings of the 2018 IEEE Custom Integrated Circuits Conference (CICC).

[27] Michel F., Steyaert M.S.J. (2012). A 250 mV 7.5 μW 61 dB SNDR SC ΔΣ Modulator Using Near-Threshold-Voltage-Biased Inverter Amplifiers in 130 nm CMOS. IEEE J. Solid-State Circuits.

